# A 14-week randomized, placebo-controlled, double-blind clinical trial to evaluate the efficacy and safety of ginseng polysaccharide (Y-75)

**DOI:** 10.1186/s12967-014-0283-1

**Published:** 2014-10-09

**Authors:** Young-Jin Cho, Hyeog-Jin Son, Kyung-Soo Kim

**Affiliations:** Department of Family Medicine, Seoul St. Mary’s Hospital, The Catholic University of Korea, Banpo-ro 222, Seocho-gu, Seoul 137-701 Republic of Korea; Department of Biomedical Engineering, Korea University College of Health Science, Anam-ro 145, Seongbuk-gu, Seoul 136-701 Republic of Korea; Clinical Research Coordinating Center, CMC, The Catholic University of Korea, Banpo-ro 222, Seocho-gu, Seoul 137-701 Republic of Korea

**Keywords:** Panax ginseng, Ginsan, Y-75, Polysaccharides, Controlled clinical trial, Immunomodulation, Natural killer cell

## Abstract

**Background:**

The Y-75 (Ginsan) acidic polysaccharide from Korean *Panax ginseng* has been shown to function as an immunomodulatory molecule. However, the efficacy of Y-75 has not been evaluated in clinical trial.

**Methods:**

We verified Y-75 (6 g/day) for safety and immune efficacy in 72 healthy volunteers aged 50–75 years using a randomized, placebo-controlled, parallel, double-blind study. The activities of natural killer (NK) cells and peripheral blood phagocytes, as well as serum levels of monocyte-derived mediators, were assessed before and after administration for 8 and 14 weeks. This trial is registered at ClinicalTrials.gov (NCT02161198).

**Results:**

Y-75 significantly enhanced NK cell cytotoxic activity by 35.2% and 40.2% from baseline after administration for 8 and 14 weeks, respectively. The phagocytic activity of peripheral blood cells was also significantly increased by 25.2% and 39.4% and serum level of TNF-α by 38.2% and 44.5% after treatment for 8 and 14 weeks, respectively. Differences in the efficacy of variables compared to the placebo group were also significant. Administration of Y-75 was well tolerated without treatment-related adverse events or alteration of complete blood cell count or blood chemistry over the entire study period.

**Conclusion:**

Y-75 was shown to be a safe and potentially effective natural alternative for enhancing immune function.

## Background

The root of *Panax ginseng* CA Meyer is an established traditional herbal medicine in Asia, having been used for thousands of years, and is now used worldwide as a nutraceutical source to improve physical performance, boost resistance against infection, reduce risk of cancer, and support current therapeutic modalities for some chronic diseases [1,2]. The biological actions of ginseng are unclear, but it appears that some of its effects are related to immunomodulation [3].

More than 100 different chemical entities of biological Interest, including ginsenosides, polysaccharides, polyacetylenes, and peptides, have been derived from ginseng species and their metabolites [4]. Among them, the polysaccharides have been shown to have effects on immunologic defense functions [5,6]. The mechanisms of action have not been fully elucidated at the molecular level, but these have been shown to activate many of the diverse types of cells in the immune system [7,8] and stimulate production of various cytokines [9-12]. A number of pharmacologically active polysaccharides with molecular weights ranging from 3500 to 160,000 Da have been identified. Importantly, these polysaccharides consist primarily of neutral sugars with various molar ratios depending on the specific ginseng of origin [4].

Ginsan is an acidic polysaccharide with a molecular weight of 150,000 Da, isolated from the aqueous Korean *Panax ginseng* extract [13,14]. Previous studies have shown that Ginsan induces the proliferation of lymphocytes [15] and maturation of bone-marrow derived dendritic cells [16,17] as well as stimulates the phagocytic activity of macrophages [18,19]. These processes include Ginsan-induced generation of cytokines, including interleukin-1β, interleukin-2, interleukin-6, interleukin-12 (IL-12), interferon-γ, and tumor necrosis factor-α (TNF-α) [14,16,18-24]. In addition, Ginsan enhances the antibody response to orally administered Salmonella antigen in a mouse model [25]. Finally, pretreatment of Ginsan protects mice from lethality induced by *Staphylococcus aureus* infection [26,27]. This survival benefit is associated with enhanced bacterial clearance from circulation and attenuation of the acute elevation of cytokines in the early septic phase, the uncontrolled response of which may be harmful to the host. Taken together, these observations strongly suggest that Ginsan mediates a significant modulatory effect on the immune system. However, the efficacy of Ginsan has not been studied in clinical trials.

Ginsan is currently available as an over-the-counter preparation and is used as a natural alternative to proactively strengthen immunity. The present study performed a randomized, placebo-controlled clinical trial to evaluate the safety and beneficial effects of Ginsan, denoted as Y-75, on immune function in a group of healthy adults aged between 50 and 75 years. The focus of this trial was modulation of innate immunity, comprising cytotoxic activity of natural killer (NK) cells, phagocytic activity of polymorphonuclear (PMN) leukocytes and mononuclear phagocytes, and serum levels of monocyte-derived mediators.

## Subjects and methods

### Participants

Volunteers were required to be in good general health and from 50 to 75 years of age. Exclusion criteria were based on patient history and included the following medical conditions: human immunodeficiency virus-1 infection and malignancy; active (currently requiring adjustment of medications) cardiovascular, renal, pulmonary, hepatic, endocrine, hematologic, neurological or psychiatric disease; recent (within 4 weeks) acute respiratory tract symptoms. Volunteers were also excluded if, at the time of enrollment, they were prescribed concurrent immunosuppressive therapy including cytotoxic agents and corticosteroids, any medication (within 4 weeks) deemed likely to interfere with the evaluation (e.g., other herbal products), or had a history of allergic or other adverse reactions to ginseng products.

### Procedures and measures

This study was conducted as a single-center, randomized, placebo-controlled, parallel, double-blind trial from September 2012 to April 2013 at Seoul St. Mary’s Hospital, The Catholic University of Korea. The study protocol, approved by the institutional review board of Seoul St. Mary’s Hospital, The Catholic University of Korea (KC12HISI0270), was conducted in accordance with the Declaration of Helsinki. Informed consent was obtained from all volunteers before entering the trial. Qualifying volunteers were randomly assigned in a 1:1 ratio to receive Y-75 at a dose of 3 g twice a day or a matching placebo. Laboratory data for safety assessment and baseline (prior to the initial dose) data for efficacy variables were obtained at screening and on the first day of investigational product administration, respectively. During scheduled visits at 8 and 14 weeks after the administration, participants were assessed for safety, efficacy, and compliance.

The primary efficacy variable was the mean percentage change in NK cell activity from a baseline level in response to Y-75. NK cells were isolated from peripheral blood of subjects at baseline, week 8 and 14 and their cytotoxic activities were measured against the K-562 tumor cell targets using a standard ^51^Cr release assay. Briefly, the target cells were labeled with 200 μCi of Na^51^CrO_4_ (PerkinElmer Life Science, USA) for 1 h, washed, and then incubated with the isolated NK cells at different effector-to-target cell ratios for 4 h. After incubation, 100 μl of the supernatant of each well was collected and measured using a Gamma counter (Cobra 2 auto gamma, Packard, Downers Grove, IL). The percentage of specific lysis was determined using the following formula: [(experimental release – spontaneous release)/(maximum release – spontaneous release)] × 100. The spontaneous and maximum releases were determined by incubating the target cells without the effectors in the medium alone or in 10% SDS, respectively. The spontaneous release was always <10% of the maximum release. Secondary efficacy variables included changes from baseline in phagocytic activity of macrophages and PMN cells from peripheral blood and serum levels of TNF-α and IL-12. The phagocytic activity was determined using pHrodo™ *E. coli* BioParticles® phagocytosis kit (Invitrogen) according to the manufacturer’s instructions. Levels of TNF-α and IL-12 in serum were determined using each ELISA kit (R&D Systems) according to the manufacturer’s instructions.

### Safety parameters

Physical examination, vital signs, and laboratory measurements for safety assessment were obtained at every visit, and participants were also asked to report any adverse events at any time. The laboratory measurements included blood chemistry (BC), complete blood count (CBC), and urinalysis. Electrocardiography was obtained at baseline and week 14. Female volunteers of childbearing age were subjected to urine human chorionic gonadotropin test at screening. Any change from the baseline status was defined as an adverse event, and the causality was assessed as definite, probable, possible, probably not related, definitely not related, and unknown. All adverse events and serious adverse events were reported according to current standards.

#### Preparation of *Y-75* or placebo

Y-75 is isolated from the ethanol-insoluble fraction of aqueous *Panax* ginseng extracts through an industrial process under strict quality control. Y-75 obtained from Dr. Yeon-Sook Yun of Health Biomed Inc. (Seoul, Korea), is composed of approximately 75% glucose, 8% galactose, 6% arabinose, 0.4% glucuronic acid, and 11% galacturonic acid. The test material was composed of freeze-dried powder of Y-75 (1 g) and starch (1 g) as a diluting agent and was packed to contain 2 g/package. The placebo used in this study was composed of caramel syrup (0.03 g) and starch (1.97 g) and was packed identically to the active treatment. Volunteers were randomly assigned to receive either Y-75 or placebo according to a randomization list generated with SAS (SAS Institute Inc., USA). Medical Excellence Inc. (Seoul, Korea), the contract research organization maintained secure copies of the treatment codes and also provided investigators with numbered sealed envelopes containing the treatment codes. The randomization codes were not decrypted until completion of the study and data analysis.

### Statistical analysis

Assuming an 8% increase (a standard deviation of 10%) in NK cell cytotoxicity from baseline over an initial 8-week treatment period of Y-75, a sample size of 29 participants per group was calculated to provide 80% power to detect an difference between Y-75 and placebo groups (two-sided α of 5%). Allowing for a dropout rate of 20%, enrollment of 72 participants was considered sufficient.

The primary efficacy analysis was performed in the intention-to-treat set and included all participants randomized with a baseline assessment. Data from study dropouts was analyzed using the last observation carried forward method. Efficacy analysis was performed with independent t-test or Wilcoxon rank sum test. Differences from baseline were analyzed with paired t-test or Wilcoxon’s signed rank test.

Safety analyses were conducted to evaluate change from baseline in the safety profile over 14 weeks. Safety data were summarized using frequencies for categorical variables and proportions as a percent. Pearson’s Chi-square test and Fisher’s extract test were used to compare the proportions of subjects reporting symptoms related to adverse events. All analyses were conducted using SAS, version 9.3 (SAS Institute Inc.) and were performed by a biostatistician.

## Results

### Participants

**Figure 1:**
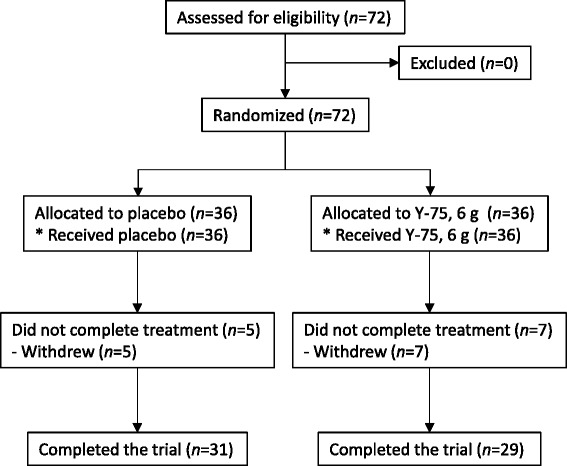
**Flow-chart of the study.**

A total of 72 randomized, healthy volunteers participated in this study and were considered for safety and intention-to-treat primary analyses. A total of 60 of the initial study subjects completed all procedures, with a dropout rate of 16.7 % comprising 12 withdrawals (7 with placebo and 5 with Y-75) (Figure 1). There was no notable difference between the two sets of statistical results, for which one was obtained from the intention-to-treat set and the other from the per-protocol set. Baseline characteristics of the participants are shown in Table 1. Characteristics were similar between the Y-75 and placebo groups. The study population had a mean age of 58.0 years and was predominantly female (97.2%).

**Table 1:** **Summary of baseline clinical characteristics**

**Characteristics**	**Y-75 (** ***n*** **= 36)**	**Placebo (** ***n*** **= 36)**
**Age, years**	57.42 ± 4.09	58.69 ± 4.34
**Female sex,** ***n*** **(%)**	34 (94.44)	36 (100)
**Weight, kg**	58.39 ± 5.42	58.83 ± 7.18
**Height, cm**	157.56 ± 4.69	158.28 ± 4.99
**Menopause,** ***n*** **(%)**	27 (87.10)	32 (94.12)
**Medical history,** ***n*** **(%)**		
Musculoskeletal and connective disease	12 33.33)	15 (41.67)
Cardiovascular disease	7 (19.44)	8 (22.22)
Metabolism and nutritional disease	5 (13.89)	6 (16.67)
Gastrointestinal disease	4 (11.11)	2 (5.56)
**Vital sign**		
Systolic BP, mmHg	109.86 ± 10.72	108.89 ± 12.25
Diastolic BP, mmHg	75.69 ± 8.12	74.31 ± 9.79
Heart rate	69.78 ± 4.85	69.56 ± 6.22
**WBC (10** ^**9**^ **/L)**	5.68 ± 1.47	5.25 ± 1.36
Neutrophils (%)	49.59 ± 11.17	49.48 ± 9.29
Lymphocytes (%)	39.63 ± 8.04	40.98 ± 8.69
Monocytes (%)	6.86 ± 1.56	6.58 ± 1.41
Eosinophils (%)	2.15 ± .55	2.46 ± 1.90
Basophils (%)	0.53 ± 0.38	0.50 ± 0.28

Data presented as mean ± SD of number (%). All differences were non-significant.

### NK cell activity

**Figure 2:**
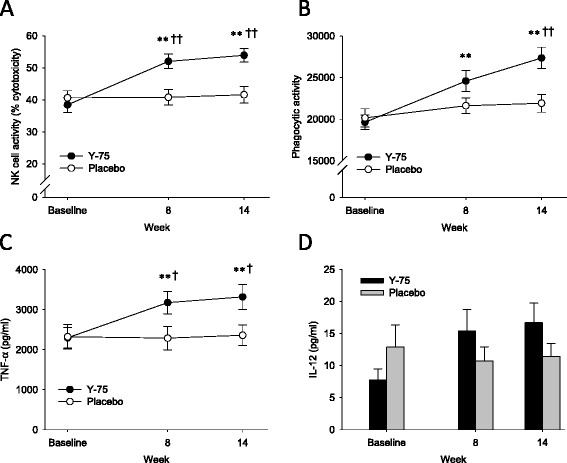
**Changes in cytotoxic activity of NK cells (A), activity of phagocytes (B), and monocyte-derived mediator (TNF-α and IL-12) levels (C, D) during the study period.** Data are mean ± SE. ** *P* <0.01 different from baseline, †*P* <0.05, †† *P* <0.01 different from the placebo group.

NK cell activity was expressed as the percentage of lysed target cells (Figure 2A). No significant differences were observed in the baseline activities between Y-75 and placebo group (38.5 ± 14.11% vs. 40.6 ± 13.4%; *P* = 0.5072). After 8 and 14 weeks of Y-75 administration, NK cell activities were significantly increased from baseline by 35.2% (*P* ≤0.0001) and 40.2% (*P* ≤0.0001), respectively. Compared with placebo, the Y-75 group exhibited a significantly higher NK cell activity at week 8 (52.0 ± 13.7% vs. 40.8 ± 14.6%; *P* = 0.0012) and week 14 (53.9 ± 13.0% vs. 41.6 ± 15.5%; *P = *0.0005).

### Phagocytic activity

The amount of FITC-labeled *E. coli* phagocytized by macrophages and PMNCs was expressed as arbitrary numbers of fluorescence intensity (Figure 2B).

Baseline phagocytic activity was 19632.1 ± 5227.1 and 20150.4 ± 6620.4 in the Y-75 and placebo groups, respectively, and increased with Y-75 from baseline by 25.2% (*P* = 0.0018) at week 8 and 39.4% (*P* ≤0.0001) at week 14. Comparing between Y-75 and placebo group, the corresponding values were 24585.3 ± 7597.2 versus 21615.4 ± 5662.6 (*P* = 0.0880) at week 8, and 27366.1 ± 7772.3 versus 21913.5 ± 6455.8 (*P* = 0.0018) at week 14.

### Monocyte-derived mediators

At baseline, serum level of TNF-α was 2297.8 ± 1551.6 pg/ml for the Y-75 and 2322.9 ± 1861.9 pg/ml for the placebo group. At week 8 and 14, Y-75 administration increased the level of TNF-α from baseline by 38.2% (*P* = 0.0006) and 44.5% (*P* = 0.0097), respectively. Significant differences between the Y-75 and placebo group were also observed at week 8 (3174.8 ± 1694.5 vs. 2286.4 ± 1769.3 pg/ml; *P* = 0.0329) and week 14 (3319.5 ± 1886.8 vs. 2360.3 ± 1552.6 pg/ml; *P* = 0.0319) (Figure 2C).

In the serum IL-12 level assay, we obtained several “undetectable” results that were subsequently excluded from statistical analysis. This resulted in a difference in the number of analyzed subjects between the scheduled visits. As a result, paired-comparison of serum IL-12 level between before and after treatments was not performed. The differences between the Y-75 and placebo groups were not significant according to Wilcoxon rank sum test, although higher levels of IL-12 were observed in the Y-75 group (Figure 2D).

### Adverse clinical events

Nineteen adverse events occurred in 9 participants given Y-75 (14 events) and 5 participants given placebo (5 events), consisting of 5 gastrointestinal problems (2 with placebo and 3 with Y-75), 4 infections (1 with placebo and 3 with Y-75), 3 complaints of musculoskeletal system pain (1 with placebo and 2 with Y-75); 2 headaches, 1 case of fatigue, and 1 foreign body aspiration in the Y-75 group; and 1 case of insomnia, 1 eye disorder, and 1 lipid metabolism disorder in the placebo group. Among those, 1 event (dyspepsia) in placebo group was classified as possibly related to the investigational drug. There was no statistical difference between the two groups for all adverse events (*P* = 0.2336), and no specific severe adverse events were observed during the study period (Table 2). Lastly, vital signs and laboratory findings for safety, which included CBC, BC, and urinalysis, were not statistically different between the two groups.

**Table 2:** **Rates of adverse events during the study period**

**Adverse event or safety variable**	**Y-75 (** ***n*** **= 36)**	**Placebo (** ***n*** **= 36)**
Volunteer with at least one adverse event, *n* (%)	9 (25.00)	5 (13.89)
Total number of adverse events	14	5
Clinical adverse event leading to discontinuation of study drug	0	0
Serious adverse event	0	0
Probably drug-related adverse event	0	1
Most common adverse events (with incidence >5% of volunteers), *n* (%)		
Gastrointestinal disease	3 (8.33)	2 (6.25)
Infection	3 (8.33)	1 (2.78)
Musculoskeletal and connective disease	2 (6.25)	1 (2.78)

## Discussion

Considerable current interest has focused on health products that are believed to enhance immunity, and ginseng is the most studied herbal medicine in this context. However, consumption of whole ginseng extract has limited clinical utility due to variations in pharmacological effects depends on preparations [2,28]. Thus, efforts have been made to purify the active components of ginseng in order to isolate its pharmacological constituents and achieve a more predictable outcome after administration. Several immunomodulatory polysaccharides have been isolated such as ginsenans [29-31], CVT-E002 [32], and Ginsan [13]. Currently, CVT-E002 is the only purified polysaccharide preparation for which safety and therapeutic effectiveness have been demonstrated in clinical trials [33-36].

In the current study, we characterized the safety and efficacy of Y-75, a novel candidate of immune modulator, in 72 healthy volunteers. Administration of Y-75 for 8 and 14 weeks safely increased NK cell activity by 35.2% and 40.2%, respectively, from baseline without altering white blood cell differential count, electrolytes, or vital signs or causing significant side effects. Importantly, compared with placebo, NK cell activity was increased by 27.6% and 29.6% at 8 and 14 weeks, respectively. Secondary efficacy variables, phagocytosis of *E. coli* by phagocytes from peripheral blood and serum level of the representative monocyte-derived mediator TNF-α, were also significantly increased by Y-75. These findings are consistent with previous preclinical laboratory studies on Ginsan.

In the current 14-week study, administration of Y-75 was well tolerated, with no discontinuation of therapy due to adverse effects. The most commonly experienced adverse events associated with usage of ginseng products include headache, sleep disturbance, and gastrointestinal disorders [37]. Long-term abuse of ginseng is associated with hypertension, gastrointestinal disturbances, insomnia, nervousness, confusion, and depression [38]. However, the number of clinical events that occurred during this trial was small, and their distribution indicated that there were no treatment-related adverse events. This high degree of safety of Y-75 was expected based on clinical literature showing that ginseng products are rarely associated with adverse effects [39].

We did not address mechanisms of action in terms of a target immune organ in the study design. Considering its high molecular weight, Y-75 is unlikely to be absorbed from the digestive tract into systemic circulation. However, in vitro evidence for gastrointestinal epithelial transfer of oligosaccharides has been reported [40]. Therefore, probable absorption of integrated molecule or digested fragment with active moiety should not be excluded. Furthermore, because the gastrointestinal tract is the largest immune organ in the body, it is interesting to speculate that this organ may be a direct target of Y-75 [41].

The pharmacokinetic profile of Y-75 after ingestion in unknown, and thus there are several limitations in this study. First, although preclinical laboratory observations showed direct stimulation of immune effector cells by Y-75, we cannot exclude the involvement of another unknown substance or substances generated through metabolism or transformation in the digestive system, as in the case of ginsenosides [42]. Second, we were also limited by uncertainty as to whether the designed dosing schedule was optimized for a primary outcome. Another limitation is gender imbalance between the groups. Male subjects (*n* = 2) were included only in Y-75 group. We re-analyzed the primary efficacy excluding the male subjects, and obtained the similar results; 51.3 ± 13.7% of NK cell activity with Y-75 versus 40.8 ± 14.6% with placebo at week 8 (*P* = 0.0028), and 53.4 ± 13.1% versus 41.6 ± 15.5% at week 14 (*P = *0.0010). However, a study including a similar number of male in the both groups is needed to exclude gender effect. Next limitation is that the findings presented in this study, did not directly reflect clinical utility as an anti-tumor or anti-septic alternative. Therefore, further investigation in larger trials designed with respect to therapeutic outcome is needed. Finally, the short study duration did not allow for prediction of the long-lasting effects on the immune system or the exclusion of emerging adverse events after long-term administration.

## Conclusions

Administration of Y-75 for 14 weeks induced augmentation in NK cell and phagocyte activities and cytokine release without affecting white blood cell differential counts in healthy volunteers. In addition, we found no significant adverse effects associated with Y-75. So far, these findings are valid for normal individuals only. However, this study provides the foundation for future clinical trials aimed at assessing the therapeutic outcomes of Y-75 as an anti-tumor or anti-septic clinical utility.

## Abbreviations

IL-12: Interleukin-12
TNF-α: Tumor necrosis factor-α
NK: Natural killer
PMN: Polymorphonuclear
FITC: Fluorescein isothiocyanate
BC: Blood chemistry
CBC: Complete blood count
